# Utility of extended HPV genotyping in cervical cancer screening: a prospective population-based cohort study in China

**DOI:** 10.3389/fonc.2025.1754438

**Published:** 2026-01-07

**Authors:** Kexin Yi, Jinyu Zhang, Hong Wang, Peipei Chen, Xingyuan Sun, Mengjie Li, Shaokai Zhang

**Affiliations:** 1 Henan Office for Cancer Control and Research, The Affiliated Cancer Hospital of Zhengzhou University & Henan Cancer Hospital, Zhengzhou, Henan, China; 2 Henan International Joint Laboratory of Cancer Prevention, Henan Engineering Research Center of Cancer Prevention and Control, Zhengzhou, Henan, China

**Keywords:** cervical cancer, China, cohort, extended HPV genotyping, screening

## Abstract

**Background:**

Cervical cancer poses a substantial global health burden, particularly in China where it contributes substantially to incidence and mortality. Conventional HR-HPV screening tests primarily target HPV16/18, limiting efficacy in regions dominated by other oncogenic genotypes and underscoring the need for extended genotyping to refine risk-stratified approaches.

**Methods:**

This prospective cohort in Henan Province, China (2017–2020), enrolled 3,299 women aged 21–64 for baseline HR-HPV testing using an extended genotyping assay detecting 14 types and identifying HPV16/18/33/52/58. HR-HPV-positives were followed annually, negatives at year 3. Three-year cumulative CIN2+/CIN3+ risks, sensitivity, specificity, PPV, and NPV were computed independent of cytology.

**Results:**

Baseline HR-HPV positivity was 18.3%. Over 3 years, 98 CIN2+ and 60 CIN3+ cases were detected. Absolute CIN2+ risks was highest for HPV16 (32.8%), comparable for HPV18/33/58 (25–28%), with relative risks exceeding 96-fold compared to HR-HPV-negatives. Extended genotyping for HPV16/18/33/52/58 improved CIN2+/CIN3+ sensitivity to 55.1% and 60.0%, respectively (vs. 51.0% and 53.3% for HPV16/18), while maintaining specificities >93%.

**Conclusions:**

In Chinese women, non-16/18 high-risk genotypes exhibit risks comparable to HPV16/18, emphasizing the value of extended genotyping in enhancing risk-based screening sensitivity and efficiency in high-burden contexts.

## Introduction

Cervical cancer represents one of the most preventable malignancies, primarily caused by persistent infection with high-risk human papillomavirus (HR-HPV) genotypes (1). Despite substantial progress in vaccination and screening, its incidence has not been fully controlled over decades. In 2022, an estimated 660,000 new cases and 350,000 deaths occurred worldwide, ranking it as the fourth most common cancer among women (2). In China, approximately 150,700 new cases and 55,700 deaths were reported that year, accounting for 22.8% of global incidence and 15.9% of mortality (3), placing the country at a high burden level internationally.

The World Health Organization (WHO) launched the Global Strategy to Accelerate the Elimination of Cervical Cancer in 2020, targeting “90-70-90” by 2030, where the “70” refers to 70% of women receiving effective screening by ages 35 and 45 (4). Current screening modalities include cytology and HR-HPV testing (5). Cytology, however, suffers from limitations such as low sensitivity, reliance on subjective interpretation, and quality control challenges (6). In contrast, HR-HPV testing provides high sensitivity and reproducibility, detecting more precancerous lesions and cancers than cytology (7). In recent years, many countries have adopted HR-HPV testing as a primary or co-testing method, with guidelines from the United States, Australia, France, the United Kingdom, India, and Brazil endorsing it for primary screening (8–14). In 2021, WHO published the second edition of its “Guidelines for Screening and Treatment of Cervical Precancerous Lesions”, which recommends HPV DNA testing as the preferred screening approach and specifies that HR-HPV assays typically detect 14 genotypes: HPV16, 18, 31, 33, 35, 39, 45, 51, 52, 56, 58, 59, 66, and 68 (15).

Globally, the predominant HR-HPV types in cervical cancer are HPV16 (55.2%), 18 (14.2%), 45 (5.0%), 33 (4.2%), and 58 (3.9%) (16). While HPV16, HPV18, and HPV45 are most oncogenic in the USA and Europe, HPV16, HPV58, and HPV52 are more prevalent in China and other Asia countries (17, 18). Risk of progression to cervical lesions varies by HR-HPV type (19, 20), with HPV31, 33, 52, and 58 posing elevated risks for low- and high-grade squamous intraepithelial lesions (LSIL/HSIL) after HPV16/18 (21, 22). This regional heterogeneity poses a challenge for standard HPV16/18-focused screening, potentially leading to suboptimal risk assessment and missed opportunities for early intervention in Asian populations. To address this, this prospective population-based cohort study evaluated the practical utility of extended genotyping in screening by assessing 3-year risks of precancerous lesions and cancer associated with different HR-HPV types in Chinese women, independent of cytology, thereby providing evidence to support its adoption for refined, risk-based screening strategies.

## Methods

### Study population and inclusion criteria

A population-based cervical cancer screening cohort was established in Jiyuan City, Henan Province, China, from April to May 2017, with participants followed for 3 years until September 2020. Eligible women were aged 21–64 years, had an intact cervix, were not pregnant or within 8 weeks postpartum, had no history of hysterectomy, cervical surgery, or cervical cancer treatment, and could provide informed consent and undergoing routine screening. The study was approved by the Institutional Ethics Committee of the Affiliated Cancer Hospital of Zhengzhou University (2017009).

### Sample collection

All eligible women underwent a gynecological examination, during which cervical samples were collected using a broom-type brush at baseline and during follow-up visits. Exfoliated cells were preserved in PreservCyt solution (Hologic Inc., Boston, USA) and stored at 4°C for subsequent liquid-based cytology and HPV DNA testing. Colposcopy was performed by experienced colposcopists. If adequate visualization was achieved during colposcopy and lesions were identified, tissue biopsy was obtained from the abnormal sites; if visualization was inadequate, endocervical curettage (ECC) was performed.

### Laboratory detection

All testing and diagnostic procedures were performed in a strictly blinded manner. HPV genotyping was conducted using HR-HPV (5 + 9) DNA Genotyping Kit (Tellgen Cor., Shanghai, China), based on multiple nucleic acid amplification polymerase chain reaction (PCR) method with fluorescence detection. This assay detects 14 HR-HPV types (16, 18, 31, 33, 35, 39, 45, 51, 52, 56, 58, 59, 66 and 68) in cervical exfoliated cells and specifically identifies HPV types 16, 18, 33, 52, and 58. Each run included positive, negative, and blank controls, along with amplification of the reference gene β-Globin to identify false negatives due to inadequate sampling or procedural errors.

Cervical cells were stained and evaluated according to The Bethesda System (TBS). Satisfactory cytological samples were classified as negative for intraepithelial lesion or malignancy (NILM), atypical squamous cells undetermined significance (ASC-US), atypical squamous cells, atypical squamous cells cannot exclude high-grade squamous intraepithelial (ASC-H), atypical glandular cells (AGC), low-grade squamous intraepithelial lesion (LSIL), high-grade squamous intraepithelial lesion (HSIL), squamous cell carcinoma (SCC), adenocarcinoma *in situ* (AIS), adenocarcinoma (ADC). NILM was considered normal liquid-based cytology (LBC). Biopsy or ECC samples were evaluated by pathologists at the Affiliated Cancer Hospital of Zhengzhou University using the cervical intraepithelial neoplasia (CIN) classification system.

### Baseline screening and follow-up procedures

At baseline, women with ASC-US or worse cytology (≥ASC-US) or HPV16/18 positivity were immediately referred for colposcopy. Those with abnormal colposcopy findings underwent biopsy and histopathological examination. A diagnosis of CIN2 or worse (CIN2+), concluding participation; otherwise, follow-up continued.

**Figure 1:**
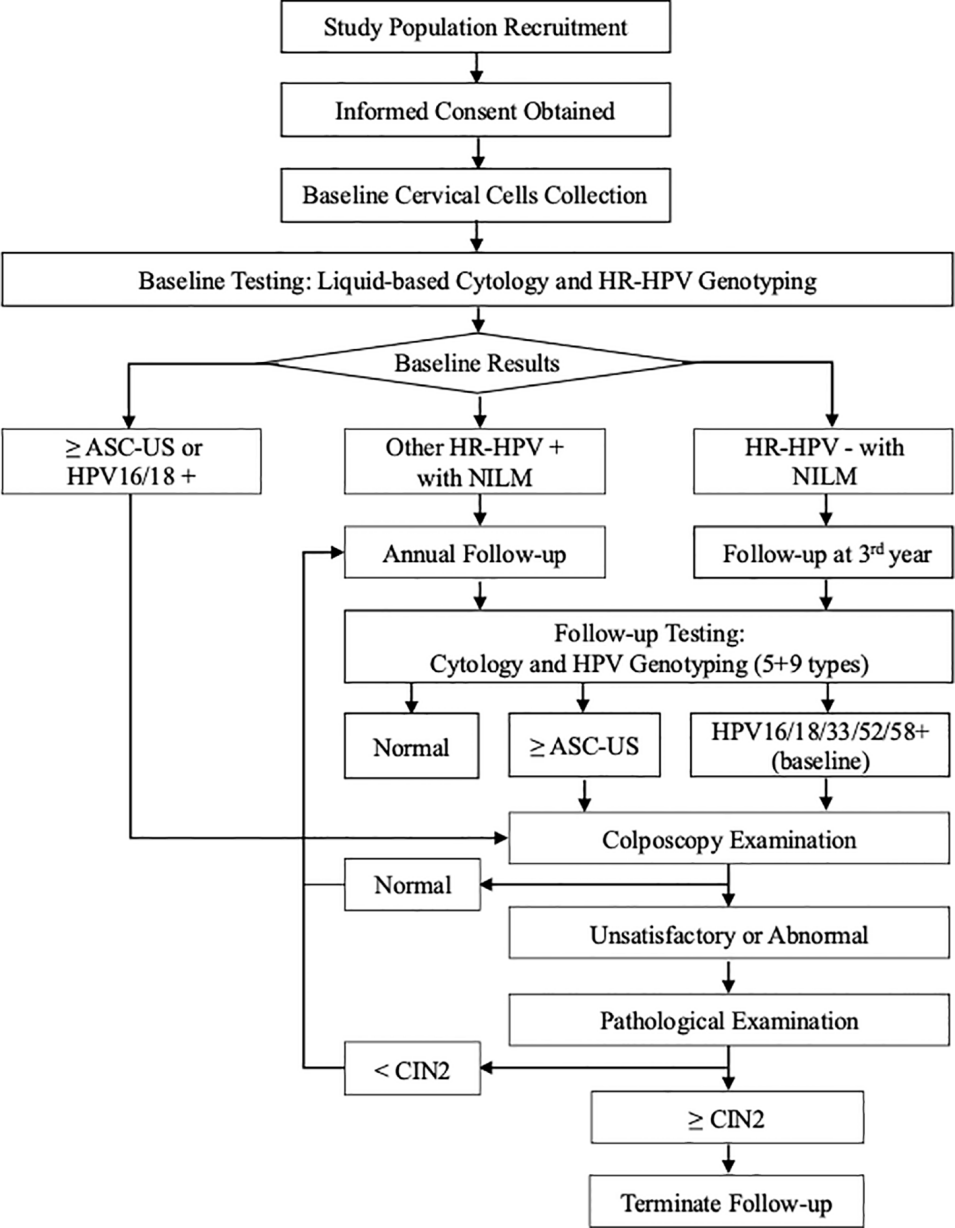
Flowchart of baseline screening and follow-up procedures. NILM, negative for intraepithelial lesion or malignancy; HPV, human papillomavirus; HR-HPV, high-risk HPV; CIN2, cervical intraepithelial neoplasia grade 2; ASC-US, atypical squamous cells undetermined significance.

HR-HPV-positive women at baseline were followed annually, while HR-HPV -negative women were reassessed at the third year. During follow-up, all participants underwent cytology and HPV DNA (5 + 9) testing. Women with normal cytology results continued to the next year visit. Women with ≥ASC-US cytology during the follow-up or baseline HPV16/18/33/52/58 positivity were referred to colposcopy, followed by biopsy and histopathological diagnosis if abnormal. CIN2+ was confirmed as the positive outcome; absence of CIN2+ by study end was deemed negative (**Figure 1**).

### Statistical analysis

Data analysis was performed using SAS version 9.4 (SAS Institute Inc., NC, USA). Continuous variables were presented as mean ± standard deviation (SD), and categorical variables as frequencies and percentages. Three-year cumulative absolute and relative risks of CIN2+ and CIN3 or worse (CIN3+), with 95% confidence intervals (95% CIs), were calculated for different baseline HR-HPV genotypes, irrespective of cytology. Sensitivity, specificity, positive predictive value (PPV), and negative predictive value (NPV) were calculated for baseline HR-HPV results in detecting cumulative CIN2+ and CIN3+ over three-year period. CIN2+ served as the primary clinical endpoint. Two-sided *P*-values ≤0.05 were considered statistically significant.

## Results

Of 4,026 eligible women enrolled in the baseline screening cohort, 3,299 (81.9%) completed the 3-year follow-up and were included in the analysis after excluding 727 (18.1%) lost to follow-up or not reaching endpoints. The mean age was 47.00 ± 9.69 years (range, 21–64 years). No significant differences were observed between completers and non-completers in baseline characteristics and HPV positivity. At baseline, HR-HPV positivity was 18.3% (604/3,299), with HPV16/18 and HPV52/58/33 being the most prevalent high-risk groups (4.8% and 8.1%, respectively), while other nine types accounted for 10.0% (**Table 1**). Over the study period, 98 CIN2+ cases and 60 CIN3+ cases were identified, with 44 CIN2+ and 29 CIN3+ at baseline, 18 CIN2+ and 16 CIN3+ during the first follow-up year, 20 CIN2+ and 9 CIN3+ during the second year, and 16 CIN2+ and 6 CIN3+ during the third year. Among CIN2+ cases, 93% (91/98) were baseline HR-HPV positive, predominantly involving HPV16/18 (51%) or HPV52/58/33 (55%), as detailed in **Table 1**.

**Table 1:** Distribution of CIN2+ and CIN3+ cases at baseline and three-year follow-up period.

Baseline HPV infection	N (%)	Baseline (n)		1^st^ year (n)		2^nd^ year (n)		3^rd^ year (n)		Overall	
		CIN2+	CIN3+	CIN2+	CIN3+	CIN2+	CIN3+	CIN2+	CIN3+	CIN2+	CIN3+
HR-HPV positive	604 (18.3)	43	29	18	16	18	8	12	6	91	59
HPV16 positive	128 (3.9)	26	20	6	6	7	2	3	1	42	29
HPV18 positive	39 (1.2)	9	5	0	0	0	0	2	1	11	6
HPV52 positive	138 (4.2)	10	7	5	4	5	2	2	1	22	14
HPV58 positive	108 (3.3)	11	6	7	6	4	2	5	2	27	16
HPV33 positive	46 (1.4)	3	3	2	2	3	1	4	3	12	9
HPV16/18 positive	159 (4.8)	32	22	6	6	7	2	5	2	50	32
HPV52/58/33 positive	266 (8.1)	22	16	13	11	11	5	8	4	54	36
Other nine types positive	329 (10.0)	15	9	5	5	12	5	5	1	37	20
HR-HPV negative	2695 (81.7)	1	0	0	0	2	1	4	0	7	1
Total	3299	44	29	18	16	20	9	16	6	98	60

HPV, human papillomavirus; HR-HPV, high-risk HPV, including HPV types 16, 18, 31, 33, 35, 39, 45, 51, 52, 56, 58, 59, 66, and 68; CIN, cervical intraepithelial neoplasia; CIN2+, cervical intraepithelial neoplasia 2 or worse; CIN3+, cervical intraepithelial neoplasia 3 or worse; HPV16/18 positive, HPV16 and/or HPV 18 is positive; HPV52/58/33, either of HPV 33, 52, 58 is positive; other nine types including HPV types 31, 35, 39, 45, 51, 56, 59, 66, 68.

Women with different baseline HPV statuses exhibited varying 3-year absolute and relative risks of CIN2 +. Absolute risks were highest for HPV16 (32.81%; 95% CI, 25.27-41.36%) and comparable among HPV18, HPV33, and HPV58 (25-28%), exceeding 15% for HPV52 and dropping to <12% for other types, with HR-HPV-negatives at 0.26%. Relative risks versus HR-HPV-negatives surpassed 96-fold for HPV16/18/33/58, while comparisons to other types showed HPV16 conferring the greatest elevation (2.92%; 95% CI, 1.97-4.32%), underscoring the disproportionate contribution of these genotypes in Chinese women (**Table 2**).

**Table 2:** The risk of CIN2+ by baseline HPV infection status.

Baseline HPV infection	Absolute risk (%, 95%CI) of CIN2+	Relative risk (%, 95%CI) of CIN2+	
		HR-HPV negative	Other nine types positive
HR-HPV positive	15.07 (12.43, 18.15)	58.00 (27.03, 124.50)	—
HPV16 positive	32.81 (25.27, 41.36)	126.30 (57.90, 275.60)	2.92 (1.97, 4.32)
HPV18 positive	28.21 (16.42, 43.90)	108.60 (44.45, 265.30)	2.51 (1.40, 4.50)
HPV52 positive	15.94 (10.70, 23.03)	61.38 (26.68, 141.20)	1.42 (0.87, 2.31)
HPV58 positive	25.00 (17.74, 33.97)	96.25 (42.87, 216.10)	2.22 (1.42, 3.47)
HPV33 positive	26.09 (15.47, 40.38)	100.40 (41.44, 243.40)	2.32 (1.31, 4.12)
HPV16/18 positive	31.45 (24.73, 39.04)	121.10 (55.80, 262.70)	2.80 (1.91, 4.09)
HPV58/52/33 positive	20.30 (15.89, 25.56)	78.16 (35.93, 170.00)	1.80 (1.23, 2.66)
Other nine types positive	11.25 (8.24, 15.15)	43.30 (19.46, 96.33)	—
HR-HPV negative	0.26 (0.11, 0.55)	—	—
Total	2.97 (2.44, 3.61)	—	—

HR-HPV, high-risk HPV, including HPV types 16, 18, 31, 33, 35, 39, 45, 51, 52, 56, 58, 59, 66, and 68; HPV16/18 positive, HPV16 and/or HPV 18 is positive; HPV52/58/33, either of HPV 33, 52, 58 is positive; other nine types including HPV types 31, 35, 39, 45, 51, 56, 59, 66, 68; 95% CI, 95% confidence interval; CIN2+, cervical intraepithelial neoplasia 2 or worse.

A similar pattern emerged for CIN3+ (**Table 3**), with absolute risks peaking at 22.66% (95% CI, 16.22-30.68%) for HPV16 and ranging 14-20% for HPV18/33/58, versus <11% for HPV52 and other types, and 0.04% (95% CI, 0.00-0.23%) for negatives. Relative to HR-HPV-negatives, risks were markedly elevated (>400-fold) for HPV16/18/33/58. Compared with other nine types positives, HPV16 again showed the greatest relative risk (3.73%; 95%CI, 2.19-6.34%), followed by HPV16/18 (3.31%; 95%CI, 1.96-5.60%), with HPV52 the lowest (1.67%; 95%CI, 0.87-3.21%) (**Table 3**), highlighting genotype-specific progression potential.

**Table 3:** The risk of CIN3+ by baseline HPV infection status.

Baseline HPV infection	Absolute risk (%, 95%CI) of CIN3+	Relative risk (%, 95%CI) of CIN3+	
		HR-HPV negative	Other nine types positive
HR-HPV positive	9.77 (7.64, 12.41)	263.30 (36.55, 1896.00)	—
HPV16 positive	22.66 (16.22, 30.68)	610.60 (83.84, 4446.00)	3.73 (2.19, 6.34)
HPV18 positive	15.38 (6.86, 30.11)	414.60 (51.12, 3363.00)	2.53 (1.08, 5.92)
HPV52 positive	10.14 (6.03, 16.42)	273.40 (36.22, 2064.00)	1.67 (0.87, 3.21)
HPV58 positive	14.81 (9.22, 22.82)	399.30 (53.44, 2983.00)	2.44 (1.31, 4.53)
HPV33 positive	19.57 (10.43, 33.39)	527.30 (68.21, 4076.00)	3.22 (1.56, 6.64)
HPV16/18 positive	20.13 (14.59, 27.07)	542.40 (74.60, 3943.00)	3.31 (1.96, 5.60)
HPV58/52/33 positive	13.53 (9.91, 18.20)	364.70 (50.21, 2649.00)	2.23 (1.32, 3.75)
Other nine types positive	6.08 (3.92, 9.25)	163.80 (22.06, 1217.00)	—
HR-HPV negative	0.04 (0.00, 0.23)	—	—
Total	1.82 (1.41, 2.34)	—	—

HR-HPV, high-risk HPV, including HPV types 16, 18, 31, 33, 35, 39, 45, 51, 52, 56, 58, 59, 66, and 68; HPV16/18 positive, HPV16 and/or HPV 18 is positive; HPV52/58/33, either of HPV 33, 52, 58 is positive; other nine types including HPV types 31, 35, 39, 45, 51, 56, 59, 66, 68; 95% CI, 95% confidence interval; CIN3+, cervical intraepithelial neoplasia 3 or worse.

Using histopathology as the gold standard, baseline HPV genotyping exhibited robust diagnostic performance for detecting cumulative 3-year CIN2+ and CIN3+ lesions (**Tables 4** and **5**). For CIN2+, overall HR-HPV positivity yielded high sensitivity (92.86%; 95%CI, 85.98-96.50%) but moderate specificity (83.97%; 95%CI, 82.66-85.20%), with near-perfect NPV of 99.74% (95%CI, 99.46-99.87%). HPV16/18 positivity balanced sensitivity (51.02%; 95%CI, 41.27-60.69%) and high specificity (96.59%; 95%CI, 95.91-97.17%), while extending to HPV52/58/33 modestly improved sensitivity (55.10%; 95%CI, 45.25-64.57%) at maintained high specificity (>93%) and NPV (>98%). For CIN3+, sensitivities were even higher (98.33% for HR-HPV; 60.00% for HPV52/58/33), with NPVs approaching 100%, supporting extended genotyping’s utility in risk stratification.

**Table 4:** The screening effect of baseline HPV genotyping for detecting cumulative CIN2+ over three years.

Baseline HPV infection	N	Sensitivity (%, 95% CI)	Specificity (%, 95% CI)	PPV (%, 95% CI)	NPV (%, 95% CI)
HR-HPV positive	604	92.86 (85.98, 96.50)	83.97 (82.66, 85.2)	15.07 (12.43, 18.14)	99.74 (99.46, 99.87)
HPV16 positive	128	42.86 (33.51, 52.74)	97.31 (96.69, 97.82)	32.81 (25.28, 41.34)	98.23 (97.71, 98.64)
HPV18 positive	39	11.22 (6.38, 18.99)	99.13 (98.74, 99.39)	28.21 (16.54, 43.78)	97.33 (96.72, 97.83)
HPV52 positive	138	22.45 (15.32, 31.66)	96.38 (95.67, 96.97)	15.94 (10.77, 22.96)	97.60 (97.00, 98.07)
HPV58 positive	108	27.55 (19.68, 37.12)	97.47 (96.87, 97.96)	25.00 (17.79, 33.93)	97.77 (97.20, 98.23)
HPV33 positive	46	12.24 (7.15, 20.19)	98.94 (98.52, 99.24)	26.09 (15.60, 40.26)	97.36 (96.75, 97.85)
HPV16/18 positive	159	51.02 (41.27, 60.69)	96.59 (95.91, 97.17)	31.45 (24.74, 39.03)	98.47 (97.98, 98.85)
HPV58/52/33 positive	266	55.10 (45.25, 64.57)	93.38 (92.46, 94.19)	20.30 (15.91, 25.54)	98.55 (98.06, 98.92)
Other nine types positive	329	37.76 (28.79, 47.64)	90.88 (89.83, 91.83)	11.25 (8.27, 15.12)	97.95 (97.37, 98.40)

HR-HPV, high-risk HPV, including HPV types 16, 18, 31, 33, 35, 39, 45, 51, 52, 56, 58, 59, 66, and 68; HPV16/18 positive, HPV16 and/or HPV 18 is positive; HPV52/58/33, either of HPV 33, 52, 58 is positive; other nine types including HPV types 31, 35, 39, 45, 51, 56, 59, 66, 68; CIN2+, cervical intraepithelial neoplasia 2 or worse; PPV, Positive Predictive Value; NPV, Negative Predictive Value.

**Table 5:** The screening effect of baseline HPV genotyping for detecting cumulative CIN3+ over three years.

Baseline HPV infection	N	Sensitivity (%, 95% CI)	Specificity (%, 95% CI)	PPV (%, 95% CI)	NPV (%, 95% CI)
HR-HPV positive	604	98.33 (91.14, 99.71)	83.84 (82.52, 85.07)	10.21 (8.00, 12.94)	99.96 (99.79, 99.99)
HPV16 positive	128	48.33 (36.17, 60.69)	97.26 (96.64, 97.77)	24.79 (17.85, 33.33)	99.02 (98.61, 99.31)
HPV18 positive	39	10.00 (4.66, 20.15)	99.10 (98.71, 99.37)	17.14 (8.10, 32.68)	98.33 (97.83, 98.72)
HPV52 positive	138	23.33 (14.44, 35.44)	96.36 (95.65, 96.95)	10.69 (6.47, 17.14)	98.54 (98.05, 98.90)
HPV58 positive	108	26.67 (17.13, 39.01)	97.38 (96.77, 97.88)	16.00 (10.10, 24.42)	98.61 (98.14, 98.96)
HPV33 positive	46	15.00 (8.10, 26.11)	98.91 (98.49, 99.22)	20.45 (11.15, 34.50)	98.42 (97.93, 98.80)
HPV16/18 positive	159	53.33 (40.89, 65.37)	96.51 (95.82, 97.09)	22.22 (16.20, 29.68)	99.10 (98.71, 99.38)
HPV58/52/33 positive	266	60.00 (47.37, 71.43)	93.27 (92.35, 94.09)	14.29 (10.50, 19.14)	99.21 (98.82, 99.47)
Other nine types positive	329	33.33 (22.73, 45.94)	90.78 (89.73, 91.73)	6.33 (4.13, 9.57)	98.65 (98.16, 99.00)

HR-HPV, high-risk HPV, including HPV types 16, 18, 31, 33, 35, 39, 45, 51, 52, 56, 58, 59, 66, and 68; HPV16/18 positive, HPV16 and/or HPV 18 is positive; HPV52/58/33, either of HPV 33, 52, 58 is positive; other nine types including HPV types 31, 35, 39, 45, 51, 56, 59, 66, 68; CIN3+, cervical intraepithelial neoplasia 3 or worse; PPV, Positive Predictive Value; NPV, Negative Predictive Value.

## Discussion

Traditional HR-HPV assays primarily target the most common oncogenic genotype, especially HPV16 and 18. However, accumulating evidence highlights the substantial oncogenic contributions of other genotypes, such as HPV31, 33, 52, and 58, particularly in Asian populations (23–25). This prospective population-based cohort study of 3,299 women in China addresses this gap by evaluating the 3-year cumulative risks of CIN2+ and CIN3+ stratified by baseline extended HPV genotyping, independent of cytology. Our findings demonstrate a distinct risk hierarchy, with HPV33 and HPV58 exhibiting risks comparable to HPV18, thereby supporting the integration of extended genotyping (HPV16/18/33/52/58) into risk-based triage to optimize screening efficiency in high-burden regions.

The 3-year absolute risks of CIN2+ were highest for HPV16 (32.81%), followed by HPV18 (28.21%), HPV33 (26.09%), and HPV58 (25.00%), with relative risks exceeding 96-fold compared to HR-HPV-negative women. This oncogenic hierarchy, where HPV33 and HPV58 rival HPV18, likely stems from genotype-specific biological factors, including higher persistence rates and enhanced E6/E7 oncoprotein activity in alpha-9/7 species, promoting transformation in host genetic backgrounds (5, 19, 20). In rural Chinese settings, cofactors like smoking may exacerbate clearance failure, as evidenced by prevalence data linking HPV58 to 15-20% of CIN2+ in high-exposure groups (17). These observations align with a recent Chinese cohort, which confirmed that HPV58 predominated in high-grade lesions among TCT-positive women, indicating shared pathways in progression (22). Conversely, Western studies like ATHENA report HPV16/18 dominating >70% of CIN3+ cases, with minimal HPV58 contribution (<5%), attributable to differences in host genetics, cofactor exposures, or viral variants (9). Our findings align closely with national and multicenter Chinese data (17) and global estimates for China (3), supporting the broader applicability of these genotype-specific risks across mainland China while acknowledging potential regional variations due to socioeconomic or environmental factors. These disparities underscore the application of extended genotyping in diverse scenarios, enabling tailored triage to reduce under-detection in Asia; future studies should incorporate persistence to refine thresholds.

Extending this stratification, baseline prevalences (HPV58: 3.3%, 27 CIN2+ cases; HPV52: 4.2%, 22 CIN2+ cases) and differential risks (HPV52: 15.94% vs. HPV58: 25.00%) highlight subtype-specific contributions, potentially amplified by co-infections that may increase progression risk through prolonged infection duration and higher viral loads, reflecting varying integration efficiencies (18). Consequently, HPV16/18 genotyping alone yielded modest sensitivities (51.0% for CIN2+, 53.3% for CIN3+), but adding HPV33/52/58 improved to 55.1% and 60.0%, respectively, with specificity >93% and enhanced PPV (20.30% for CIN2+), optimizing triage in resource-strained programs. A study of Yunnan Province complements this by linking HPV33 to E6/E7 mRNA expression in biopsies, a marker of active carcinogenesis, advocating hybrid cytology-genotyping models (21). However, another analysis in Sichuan revealed higher HPV52 burdens in unscreened groups, indicating access disparities that could affect outcomes (26). Thus, extended genotyping optimizes algorithms by reducing unnecessary colposcopies (potentially by 20-30%) (27), addressing resource strain in China’s rural programs, particularly in self-sampling contexts where cytology uptake varies.

From a public health perspective, our results amid China’s low vaccine coverage (28, 29) highlight the rising relevance of non-HPV16/18 high-risk types (HPV52/58/33 at 8.1% prevalence, 54 CIN2+ cases), which may be further amplified by potential type replacement effects post-vaccination, such as increased circulation of non-vaccine types like HPV52/58 observed in cross-sectional studies following vaccine introduction. Integrating extended genotyping with China’s expanding vaccination programs, could enable real-time monitoring of vaccine impact, refine risk-stratified screening intervals for vaccinated women, and mitigate resurgence in high-burden areas, thereby supporting WHO’s 90-70–90 targets (3, 4) through adaptive, evidence-based strategies. The lower HPV52 risk relative to HPV58 suggests tailored monitoring intervals, enabling sustainable screening in high-burden areas like Henan. This is corroborated by a 2025 survey of 24,588 rural women, where HR-HPV genotyping guided vaccination integration to mitigate disparities (30). Integrating extended genotyping into guidelines could enhance equity and early intervention, especially in resource-limited settings; longitudinal epidemiological studies should track post-vaccination dynamics to prevent resurgence and inform adaptive strategies.

This study’s strengths include prospective design, large sample size, blinded assessments, and >80% follow-up retention, yielding robust risk estimates aligned with real-world Chinese screening. The cohort’s genotype distribution mirrors national patterns, enhancing generalizability, while CIN2+ as the endpoint ensures clinical relevance. The study also has several limitations. First, the single-center design may overlook regional variations. Second, the lack of genotype persistence data risks overestimating risks from transient infections, however, which will be incorporated in subsequent analysis. Furthermore, the 18.1% loss to follow-up rate may introduce selection bias. However, the lack of differences in baseline characteristics between completers and non-completers supports the robustness of our risk estimates. Future work should extend follow-up, establish a dynamic cohort, and expand the study regions to provide more robust evidence.

In conclusion, this study provides longitudinal evidence of genotype-specific risks in Chinese women, advocating extended HPV genotyping for refined screening algorithms and accelerated elimination efforts in evolving epidemiological contexts.

## Data Availability

The original contributions presented in the study are included in the article/supplementary material. Further inquiries can be directed to the corresponding author.
